# Predictors of mortality in patients with acute small-bowel perforation transferred to ICU after emergency surgery: a single-centre retrospective cohort study

**DOI:** 10.1093/gastro/goab054

**Published:** 2021-12-28

**Authors:** Jianzhang Wu, Ping Shu, Hongyong He, Haojie Li, Zhaoqing Tang, Yihong Sun, Fenglin Liu

**Affiliations:** Department of General Surgery, Zhongshan Hospital, Fudan University, Shanghai, P. R. China

**Keywords:** small-bowel perforation, blood lactate, APACHE-II score, malignant tumour

## Abstract

**Background:**

Although small-bowel perforation is a life-threatening emergency even after immediate surgical intervention, studies have rarely investigated surgical outcomes due to its relatively low incidence. This study aimed to investigate the outcomes of emergency surgery for patients with small-bowel perforation transferred to the intensive care unit (ICU) and the risk factors for mortality.

**Methods:**

Consecutive patients with small-bowel perforation who were confirmed via emergency surgery and transferred to the ICU in Zhongshan Hospital, Fudan University (Shanghai, China) between February 2011 and May 2020 were retrospectively analysed. Medical records were reviewed to determine clinical features, laboratory indicators, surgical findings, and pathology.

**Results:**

A total of 104 patients were included in this study, among whom 18 (17.3%), 59 (56.7%), and 27 (26.0%) underwent perforation repair, segmental resection with primary anastomosis, and small-bowel ostomy, respectively. Malignant tumours were the leading cause of perforation in these patients (40.4%, 42/104). The overall post-operative complication rate and mortality rates were 74.0% (77/104) and 19.2% (20/104), respectively. Malignant tumour-related perforation (odds ratio [OR], 4.659; 95% confidence interval [CI], 1.269–17.105; *P* = 0.020) and high post-operative arterial blood-lactate level (OR, 1.479; 95% CI, 1.027–2.131; *P* = 0.036) were identified as independent risk factors for post-operative mortality in patients with small-bowel perforation transferred to the ICU.

**Conclusions:**

Patients with small-bowel perforation who are transferred to the ICU after emergency surgery face a high risk of post-operative complications and mortality. Moreover, those patients with malignant tumour-related perforation and higher post-operative blood-lactate levels have poor prognosis.

## Introduction

Gastrointestinal (GI) perforation is a common surgical emergency that carries substantial morbidity and mortality [1–3]. Initially, patients often experience a sudden onset of abdominal pain, while severe patients may develop septic-shock symptoms secondary to peritonitis, such as consciousness disorder, abnormal body temperature, hypotension, and tachycardia [4, 5]. Compared with perforations at other sites throughout the GI tract, small-bowel perforations are uncommon and often display atypical clinical manifestations [6, 7]. However, severe or even life-threatening infections are more likely to occur with small-bowel perforation [8, 9].

Various factors can cause small-bowel perforation, with its aetiology spectrum varying geographically and economically. Reports have found mechanical obstruction and immune-mediated disease (Crohn’s disease) to be the leading causes in Western countries [2, 10, 11], whereas small-bowel perforations secondary to typhoid and tuberculosis were more common in developing countries [6, 8, 12]. Although several studies have indicated that colorectal perforation secondary to cancer and infection carry high mortality rates [13–15], few have explored the association between the aetiology of small-bowel perforation and its prognosis.

Many existing studies focus on the prognosis of colorectal or intestinal perforation [14, 16]. Patients with small-bowel perforation are rarely studied as a single group due to its relatively low incidence and insufficient sample size. One study had reported that small-bowel perforation was associated with considerable morbidity and mortality while also identifying prognostic factors associated with the same [17]. However, these prognosis-related clinical indicators and scoring systems have only been validated in small study populations. Additionally, clinical practice has focused on the subgroup of patients admitted to the intensive care unit (ICU) who exhibit adverse clinical outcomes due to their poor general condition and numerous potential risk factors [18]. Hence, accurate evaluation of ICU patients has been a hot issue for clinicians [19]. Nonetheless, whether previously validated predictors are applicable for ICU patients who had undergone emergency surgery remains to be explored.

This study retrospectively analysed ICU patients who underwent emergency surgery for small-bowel perforation at our centre to describe clinical characteristics and surgical outcomes, and identify the prognostic factors associated with post-operative mortality.

## Patients and methods

### Study design and patients

From 1 February 2011 to 30 May 2020, consecutive patients who underwent emergency operations for GI perforation in Zhongshan Hospital, Fudan University (Shanghai, China) were retrospectively screened. The inclusion criteria were patients who underwent urgent surgery for GI perforation precisely identified during the surgery. The exclusion criteria were as follows: (i) upper GI or colorectal perforation; (ii) perforation of the appendix; (iii) simultaneous non-small-bowel perforation; (iv) anastomotic leakage; (v) patients who were not transferred to the ICU; (vi) lack of medical data.

The diagnosis was established based on abdominal physical examination and clinical assessment with the aid of abdominal and pelvic computed tomography scanning. Once the patient presented signs of peritonitis and free air in the peritoneal cavity, the surgeon considered exploratory laparotomy. Prior to the surgery, all patients were required to undergo fasting, nasogastric decompression, establishment of intravenous access, broad-spectrum antibiotic administration, and fluid resuscitation. Once the site of perforation was identified during exploratory laparotomy, the surgical procedure and necessity for ostomy were dependent on the intraoperative evaluation of the surgeon. Abdominal lavage with copious saline solution was routinely performed regardless of the severity of the peritoneal contamination. Patients with any one of the unstable conditions below were transferred to the ICU for further vital-sign monitoring and supportive treatment: (i) severe abdominal infection with diffuse fecal ascites; (ii) vasopressor requirement to maintain a mean arterial pressure of ≥65 mmHg during the preoperative and intraoperative periods; (iii) requiring prolonged mechanical ventilation with oxygenation index <300; or (iv) the occurrence of single or multiple organ(s) dysfunction.

### Outcome and clinical details

Clinical features, laboratory indexes, surgical findings, and pathology were reviewed based on the medical records. The data collected included sex, age, American Society of Anesthesiologists (ASA) score, Acute Physiology and Chronic Health Evaluation II (APACHE-II) scores, white blood cell (WBC) count, and procalcitonin (PCT) and lactate levels. To minimize treatment interference, WBC counts and PCT levels were determined from the most recent blood tests before surgery, whereas lactate levels were determined immediately after ICU admission following surgery. Surgical records and post-operative pathology were used to identify the aetiology of the small-bowel perforation and surgical procedure used for its management.

The primary outcome was death from any cause after surgery, whereas the secondary outcome included post-operative complications classified and graded according to the Clavien–Dindo classification [20, 21]. For patients with multiple complications, the highest Clavien–Dindo grade was identified as the final complication grade. Mortality was defined as death after a single admission or within 30 days of surgery. The use of patients’ clinical data was approved by the Clinical Research Ethics Committee of Zhongshan Hospital, Fudan University (B2020-350) and this study was performed in accordance with the ethical standards presented in the 1964 Declaration of Helsinki and its later amendments.

### Statistical analysis

Statistical analysis was performed using SPSS 25.0. The *t*-test and Mann–Whitney *U* test were used to analyses quantitative data with normal and non-normal distribution, respectively, whereas Pearson's chi-squared test or Fisher's exact test were performed to analyses classified data. Multivariate analysis using the binary logistic-regression model was utilized to identify risk factors for post-operative mortality. Statistical significance was set at an α level of 0.05.

## Results

### Clinical and surgical characteristics

**Figure 1.**
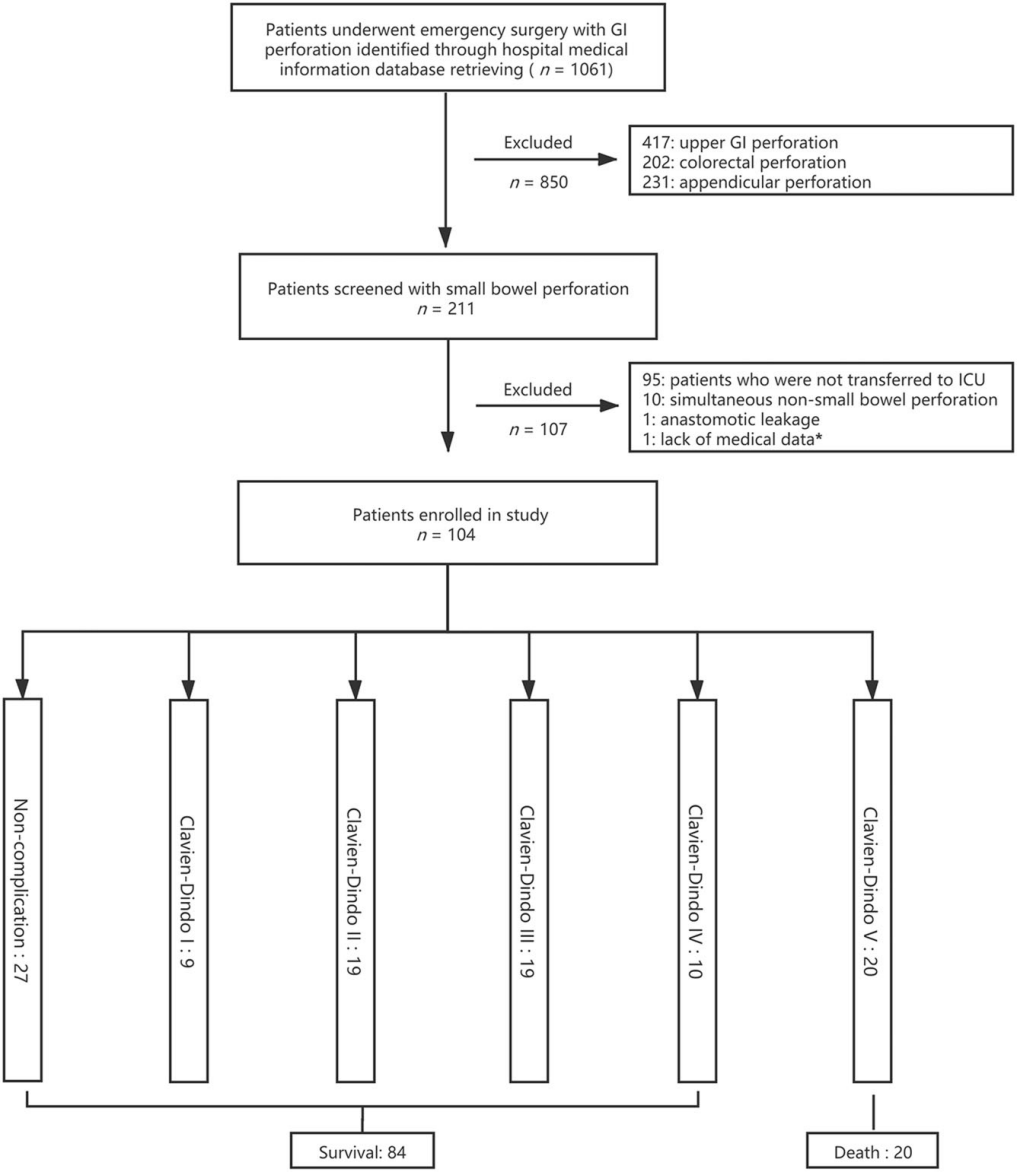
The flow chart of the study. GI, gastrointestinal; ICU, intensive care unit. *The medical information was sealed up due to medical dispute.

A total of 1,061 consecutive patients with GI perforation who underwent emergency surgery in Zhongshan Hospital, Fudan University (Shanghai, China) between 1 February 2011 and 30 May 2020 were identified and screened based on the eligible criteria (Figure 1). Finally, a total of 104 small-bowel-perforation cases were enrolled and analysed.

Among the 104 patients included in this study, 68 were male and 36 were female (1.89:1) with a mean age of 64.7 ± 15.6 years (Table 1). Moreover, 40.4% (42/104) of the patients were admitted to the ICU for malignant tumour-related small-bowel perforation. Before surgery, 44 cases (42.3%) had an ASA grade of 1–2, whereas the other 60 cases had an ASA grade of ≥3. According to the diagnostic criteria for systemic inflammatory response syndrome (SIRS), 59.6% (62/104) of the patients had abnormal preoperative leucocyte levels. Perforation repair (including repair of the bowel wall defect after wedge resection of the diverticulum with perforation, without segmental resection) was performed in 18 patients (17.3%), segmental resection with primary anastomosis was performed in 59 patients (56.7%), and small-bowel ostomy was performed in the remaining 27 patients (26.0%). Based on ICU monitoring data, the study population was determined to have a median APACHE-II score of 14.50, with a median post-operative lactate level of 1.92 mmol/L. The median length of post-operative hospital stay was 12.3 days.

**Table 1.** Clinical characteristics and outcomes of 104 patients in the study

Characteristic	Value
Age, year, mean ± SD	64.7 ± 15.6
Male, *n* (%)	68 (65.4%)
Aetiology, *n* (%)	
Malignant tumour	42 (40.4%)
Lymphoma	24 (23.1%)
Metastatic tumour	17 (16.3%)
Stromal tumour	1 (1.0%)
Bowel obstruction	21 (20.2%)
Adhesions	13 (12.5%)
Strangulated hernia	6 (5.8%)
Phytobezoar	2 (1.9%)
Foreign-body ingestion	13 (12.5%)
Trauma	7 (6.7%)
Intestinal ischaemia	5 (4.8%)
Diverticulum	4 (3.8%)
Idiopathic	4 (3.8%)
Crohn’s disease	3 (2.9%)
Iatrogenic	3 (2.9%)
Intestinal tuberculosis	2 (1.9%)
ASA grade, *n* (%)	
Low (1–2)	44 (42.3%)
High (≥3)	60 (57.7%)
WBC count (×10^9^/L), *n* (%)	
>12 or <4.0	62 (59.6%)
≤12 and ≥4.0	42 (40.4%)
Lactate, mmol/L, median (IQR)	1.92 (1.32–2.87)
PCT^a^, ng/mL, median (IQR)	2.92 (0.34–12.44)
APACHE-II score, median (IQR)	14.50 (8.00–18.27)
Procedure, *n* (%)	
Perforation repair	18 (17.3%)
Segmental resection with primary anastomosis	59 (56.7%)
Small-bowel ostomy	27 (26.0%)
Hospital stay^b^, days, median (IQR)	12.3 (8.7–20.5)
Clavien–Dindo grade, *n* (%)	
No complication	27 (26.0%)
I	9 (8.7%)
II	19 (18.3%)
III	19 (18.3%)
IV	10 (9.6%)
V (Death)	20 (19.2%)

APACHE-II, Acute Physiology and Chronic Health Evaluation II; ASA, American Society of Anesthesiologists; IQR, interquartile range; PCT, procalcitonin; SD, standard deviation; WBC, white blood cell.

^a^ The missing proportion of PCT was 27.9% (29/104).

^b^ Duration of hospitalization after surgery.

### Aetiologies of perforation

**Figure 2.**
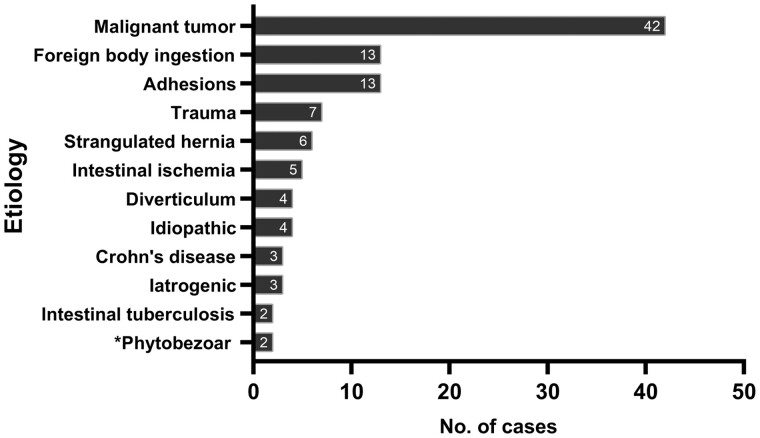
The spectrum of aetiologies responsible for small-bowel perforation ranking by the number of cases. *Two patients developed phytobezoar at the beginning of the ascending colon, resulting in perforation secondary to small-bowel obstruction.

The aetiologies of perforation were determined based on surgical findings and post-operative pathological results. In four cases of idiopathic perforation, no aetiological factors were apparent from the biopsy specimen of intestinal perforation. All other cases had precise aetiological diagnoses, which were subsequently ranked according to the number of cases in Figure 2.

Malignant tumour-related perforation was the most common cause in patients transferred to the ICU (42/104, 40.4%), among whom 24 suffered from lymphoma, 17 suffered from secondary cancer, and the remaining case suffered from stromal tumour (Table 1). Lymphoma (24/42, 57.1%) was the most common subtype of malignant tumour, most of which were the aggressive histopathologic types with high Ki-67 expression. Monomorphic epitheliotropic intestinal T-cell lymphoma and diffuse large B-cell lymphoma were the two most common pathologic types of lymphomas (Supplementary Table 1). Among the 24 patients with small-bowel perforation secondary to intestinal lymphoma, 9 were definitely diagnosed with lymphoma prior to the perforation, whereas the remaining 15 were verified based on post-operative biopsy pathology. Secondary cancer (17/42, 40.5%, 13 from other abdominal organs and 4 from the lungs) and stromal tumour (1/42, 2.4%) were the other two subtypes of malignant tumours responsible for perforations.

### Complication rate and mortality

Among the 104 patients, 77 (74.0%) had at least one post-operative complication. Respiratory complications (38/104, 36.5%), intra-abdominal infection (32/104, 30.8%), and surgical-site complications (including fat necrosis, incision infection, incision split, and drain-site infection) (23/104, 22.1%) were the three most common post-operative complications. Respiratory complications such as pneumonia, pleural effusion, and prolonged respiratory failure requiring ventilator support were noted in 7 (6.7%), 20 (19.2%), and 11 (10.6%) patients, respectively. Post-operative complications were classified according to the Clavien–Dindo classification. Accordingly, 9, 19, 19, 10, and 20 cases were classified as having grade I, II, III, IV, and V complications, respectively (Figure 1).

**Figure 3.**
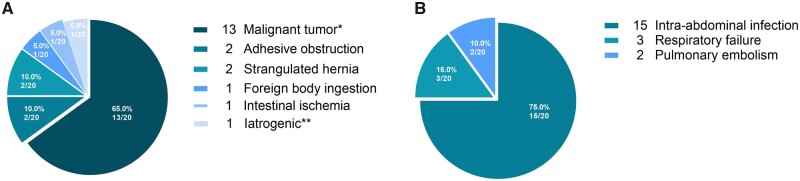
Distribution of aetiology and direct causes of death in the death group. (A) Distribution of aetiology. (B) Distribution of direct causes of death. *The subgroup of malignant tumour includes nine patients with lymphoma and four patients with secondary tumours. **Iatrogenic perforation secondary to radical resection of left renal carcinoma.

The overall mortality rate was 19.2% (20/104). Deaths from malignant tumour-related perforations accounted for 65.0% (13/20) of the total deaths, followed by adhesive obstruction (2/20, 10.0%), strangulated hernia (2/20, 10.0%), intestinal ischaemia (1/20, 5.0%), foreign-body ingestion (1/20, 5.0%), and iatrogenic perforation secondary to radical resection of left renal carcinoma (1/20, 5.0%) (Figure 3A). Septic shock secondary to intra-abdominal infection, respiratory failure, and pulmonary embolism were the direct causes of death (Figure 3B).

### Risk factors for mortality in ICU patients

**Figure 4.**
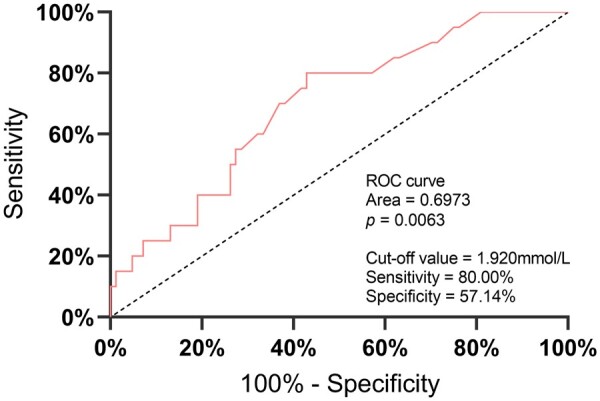
The receiver-operating characteristic (ROC) curve of post-operative blood lactate. Lactate was an independent risk factor for post-operative mortality, with a cut-off value of 1.920 mmol/L.

A total of 20 ICU patients (19.2%) died within 1 month after emergency surgery. Table 2 summarizes the factors associated with post-operative mortality. Our statistical analysis discovered that the mortality risk for patients with malignant tumour-associated perforations were significantly higher than those without malignant tumour-associated perforations (*P* = 0.013). Moreover, compared with the 84 surviving patients, the 20 patients who died had significantly higher post-operative arterial blood-lactate levels (2.53 [IQR, 1.96–4.00] vs 1.70 [IQR, 1.22–2.68], *P* = 0.005) and APACHE-II scores (18.00 [IQR, 13.97–23.75] vs 13.00 [IQR, 7.01–18.00], *P* = 0.012). Multivariate regression analysis identified malignant tumour (OR, 4.659; 95% CI, 1.269–17.105; *P* = 0.020) and a high blood-lactate level (OR, 1.479; 95% CI, 1.027–2.131; *P* = 0.036) as independent risk factors for post-operative death. Furthermore, receiver-operating characteristic (ROC) curves were constructed for lactate levels as a predictor of mortality (Figure 4), which subsequently showed 1.920 mmol/L as the best cut-off value, with a sensitivity of 80.00% and a specificity of 57.14% for predicting death. The area under the ROC curve was 0.6973.

**Table 2.** Univariate and multivariate analysis of clinical and laboratory data between death and survivor groups

Factor	Survival	Death	*P*-value	Multivariate analysis	
	(*n* = 84)	(*n* = 20)		Odds ratio (95% CI)	*P*-value
Gender			0.126		0.068
Male	52	16		Reference	
Female	32	4		0.232 (0.048–1.115)	
Age, year, mean ± SD	64.2 ± 16.2	66.6 ± 12.7	0.541	1.022 (0.978–1.069)	0.330
ASA			0.081		0.198
Low (1–2)	39	5		Reference	
High (≥3)	45	15		2.365 (0.638–8.768)	
WBC count (×10^9^/L)			0.329		0.204
>12 or <4.0	52	10		0.472 (0.148–1.505)	
≤12 and ≥4.0	32	10		Reference	
Malignant tumour (yes/no)	29/55	13/7	0.013	4.659 (1.269–17.105)	0.020
Blood lactate, mmol/L, median (IQR)	1.70 (1.22–2.68)	2.53 (1.96–4.00)	0.005	1.479 (1.027–2.131)	0.036
APACHE-II score, median (IQR)	13.00 (7.01–18.00)	18.00 (13.97–23.75)	0.012	1.018 (0.937–1.106)	0.676
Procedure			0.131		0.883
Perforation repair	17	1		Reference	
Segmental resection with primary anastomosis	48	11		1.045 (0.097–11.231)	0.971
Small-bowel ostomy	19	8		1.431 (0.115–17.812)	0.780
PCT, ng/mL, median (IQR)	2.20 (0.28–7.89)	5.46 (0.98–39.83)	0.155^a^		

APACHE-II, Acute Physiology and Chronic Health Evaluation II; ASA, American Society of Anesthesiologists; CI, confidence interval; IQR, interquartile range; PCT, procalcitonin; SD, standard deviation; WBC, white blood cell.

^a^ PCT was not included in the multivariate regression analysis due to the missing proportion of 27.9% (29/104).

## Discussion

The current study found an overall complication rate and mortality of 74.0% and 19.2% for patients with small-bowel perforation who were transferred to the ICU after surgery, respectively, which were close to the research data reported by Tan *et al*. (76.5% and 19.1%) [17]. The aforementioned results indicated that small-bowel perforation was a critical surgical emergency that carried a relatively high complication rate and mortality rate despite advances in medical technology and rigorous medical care. Moreover, our findings showed that the APACHE-II score, malignant tumour, and post-operative blood-lactate level were associated with poorer clinical outcomes, with the presence of malignant tumour and a higher post-operative blood-lactate level independent predictors of post-operative mortality in ICU patients with small-bowel perforation.

Data from the present study suggest that a wide spectrum of aetiologies is responsible for small-bowel perforation, malignant tumours (40.4%) being the leading aetiologies. According to previous domestic statistics in China, adenocarcinomas (52.9%) and stromal tumours (33.6%) were the most common primary tumours of the small bowel [22]. However, no case of adenocarcinoma-related perforation had been found herein, while only one case (1/42) had stromal tumour. Previous studies have reported that perforation was the most common complication in lymphoma cases, accounting for >25% [23]. Therefore, it can be reasonably assumed that patients with small-intestinal lymphoma have a greater risk of perforation compared with those with other primary tumours in the small bowel. Vaidya *et al.’*s research suggested that most lymphomas originating from the small bowel were B-cell type, whereas only 10%–25% were T-cell type carrying poorer prognosis [24]. On the contrary, the current study showed that T-cell lymphoma (14/24, 58.3%) was more common than B-cell lymphoma (10/24, 41.7%) in patients with perforated small-intestinal lymphoma. Furthermore, 37.5% (9/24) of the patients with lymphoma died after surgery, resulting in a mortality rate similar to that obtained in Vaidya’s study (30.4%) [24]. The aforementioned results indicated that patients with small-bowel perforation secondary to intestinal lymphoma were more likely to experience a worse prognosis. However, the prognostic differences of distinct clinicopathologic types in small-bowel lymphoma need to be further explored in a larger-sample study. Among the patients with perforation secondary to lymphoma, five received chemotherapy for lymphoma within 3 months before surgery, while four had a history of steroid hormone use. Although the poor prognosis was associated with an immune disorder caused by the tumour itself, antitumour therapy can aggravate immunodeficiency when confronted with perforation and subsequent infection [24, 25]. No perforation secondary to typhoid fever was discovered and only two patients were diagnosed with tuberculosis infection, which are the most common aetiologies in developing countries, including China [26]. According to the medical-information database, a reasonable explanation might be that the vast majority of the study population was from economically developed areas in eastern China.

Furthermore, our study demonstrated that the lung was the most common primary site of metastatic tumours causing small-bowel perforation. Some researchers suspected that the perforations might be associated with targeted therapies for lung cancer. In fact, among four of our cases with metastatic tumour secondary to lung cancer, three developed perforation immediately after targeted therapy, among whom two received bevacizumab and one received afatinib. Coincidentally, among the five patients with lymphoma who received chemotherapy prior to perforation, three received rituximab and two received gemcitabine and oxaliplatin. These targeted/chemotherapeutic drugs could inhibit tumour angiogenesis, subsequently leading to tumour necrosis [27]. They could also regulate the signalling pathways of tumour cells, causing their apoptosis [28]. These effects would make the lesions prone to perforation. Recently, targeted therapy has also been reported to cause bowel perforation in metastatic lesions from different primary sites [29].

The current study showed that mortality was mainly due to sepsis caused by severe intra-abdominal infections (Figure 3B)—a finding that has been verified in other studies [16, 30]. Sepsis was thought to be the SIRS of the body induced by infection and the conception has been termed sepsis version 1.0 [31]. The criteria for SIRS include four indicators: body temperature, heart rate, respiratory status, and WBC count [4]. Given the lack of clinical information, we only grouped our patients according to their WBC count based on the SIRS criteria. However, our analysis did not identify WBC count as a significant factor associated with post-operative mortality in patients with small-bowel perforation (Table 2). Clinical practice has shown that the SIRS criteria are too sensitive and the diagnosis of sepsis 1.0 is highly heterogeneous [32]. The Sequential Organ Failure Assessment (SOFA) scoring system has been adopted to define sepsis 3.0 by placing emphasis on organ functions and host response to infection [31]. The SOFA scores have been found to have higher prognostic accuracy for patients, especially for those admitted to the ICU, compared with the SIRS criteria [33]. However, SOFA scoring has not been widely utilized in ICU patients at our hospital and the validity of SOFA for evaluating the severity of these patients needs further exploration. APACHE-II has been utilized in clinical practice earlier than the SOFA scoring system and is currently widely used for the classification and prognostic prediction of critically ill patients. Horiuchi *et al*. found that APACHE-II scores were closely associated with prognosis, with patients having APACHE-II scores of ≥20 exhibiting significantly increased mortality rates [34]. The current study found that, among in the patients admitted to the ICU after surgery, those non-survivors had significantly higher median APACHE-II scores compared with survivors (18.00 [IQR, 13.97–23.75] vs 13.00 [IQR, 7.01–18.00], *P* = 0.012). Nevertheless, multivariate regression analysis did not identify the APACHE-II score as an independent predictor of mortality.

As an excellent indicator reflecting the state of tissue oxygenation and metabolism, blood-lactate levels have attracted increasing attention. Sepsis 3.0 defines septic shock as a condition requiring vasopressor therapy to maintain a mean arterial pressure of ≥65 mmHg and blood-lactate level of >2 mmol/L after appropriate fluid replacement [31]. Previous studies have shown that post-operative arterial blood-lactate levels were associated with mortality in patients with colorectal perforation [35]. Indeed, our findings showed that survivors had significantly lower arterial blood-lactate levels than non-survivors (1.70 vs 2.53, *P* = 0.005). Furthermore, our analysis identified lactic acid as an independent risk factor for mortality, with a cut-off value of 1.920 mmol/L based on ROC curve analysis (Figure 4).

Recent years have witnessed the extensive clinical application of serum PCT. Indeed, serum PCT levels have been found to increase with the severity of infection and organ dysfunction [36, 37]. Multiple studies have shown that PCT is a prognostic indicator [38] and that PCT-guided therapies may predict treatment response and reduce the length of antibiotic treatments in patients with severe intra-abdominal infection [39, 40]. PCT has been suggested to be one of the central node molecules in sepsis that plays an important role in the interaction between cytokine networks and other molecular interactions [41]. However, univariate analysis conducted herein did not find an association between PCT levels and mortality. Considering the high proportion of missing data (27.9%, 29/104) in the ICU group, the clinical utility of serum PCT requires further study.

Given that the cases and data included in the current study were obtained from a single centre with a limited sample size, the included population may have different clinical characteristics from the overall population. Given the nature of our single-centre retrospective study, selection bias was unavoidable, which may have affected the statistical results. Data for some important clinical indicators such as PCT were incomplete or missing. Additionally, since we focused on the prognosis analysis of ICU patients, whether the consequence is applicable for all small-bowel-perforation patients remains to be further explored. To provide more reliable and accurate evidence-based medical evidence, prospective multicentre studies are required.

The present study demonstrated that ICU patients with small-bowel perforation exhibited a high complication rate and mortality rate after emergency surgery. The presence of malignant tumours, which were the leading cause of perforation among those admitted to the ICU, was identified as an independent risk factor for post-operative mortality. Moreover, lactate was identified as another independent prognostic indicator in patients transferred to the ICU, with a post-operative lactic-acid level of >1.920 mmol/L requiring special attention and medical care.

## Supplementary Data

Supplementary data is available at *Gastroenterology Report* online.

## Authors’ Contributions

F.L. and Y.S. had full access to all study data and took responsibility for the integrity of the data and accuracy of data analysis. P.S., H.H., and F.L. designed the experimental flow of the study. J.W., P.S., and H.H. collected the data and were major contributors in the drafting of the manuscript. J.W., Z.T., and H.L. analysed and interpreted the patient data. H.H., Y.S., and F.L. revised the manuscript. All authors read and approved the final manuscript.

## Funding

This work was supported by National Nature Science Foundation of China (No. 82172803).

## Supplementary Material

goab054_Supplementary_Data

## References

[1] Søreide K , Thorsen K, Harrison EM et al Perforated peptic ulcer. Lancet 2015;386:1288–98. 26460663 10.1016/S0140-6736(15)00276-7 PMC4618390

[2] Chaikof EL. Nontraumatic perforation of the small bowel. Am J Surg 1987;153:355–8. 3565679 10.1016/0002-9610(87)90576-9

[3] Bielecki K , Kamiński P, Klukowski M. Large bowel perforation: morbidity and mortality. Tech Coloproctol 2002;6:177–82. 12525912 10.1007/s101510200039

[4] Bone RC , Balk RA, Cerra FB et al Definitions for sepsis and organ failure and guidelines for the use of innovative therapies in sepsis. The ACCP/SCCM Consensus Conference Committee. ACCP/SCCM. Chest. 1992;101:1644–55. 1303622 10.1378/chest.101.6.1644

[5] Sigmon DF , Tuma F, Kamel BG et al Gastric perforation. In: StatPearls. Treasure Island, FL: StatPearls Publishing, 2021. https://www.ncbi.nlm.nih.gov/books/NBK519554/ (1 July 2021, date last accessed). 30137838

[6] Eid HO , Hefny AF, Joshi S et al Non-traumatic perforation of the small bowel. Afr Health Sci 2008;8:36–9. 19357730 PMC2408541

[7] Lo Re G , Mantia FL, Picone D et al Small bowel perforations: what the radiologist needs to know. Semin Ultrasound CT MR 2016;37:23–30. 26827735 10.1053/j.sult.2015.11.001

[8] Jain BK , Arora H, Srivastava UK et al Insight into the management of non-traumatic perforation of the small intestine. J Infect Dev Ctries 2010;4:650–4. 21045358 10.3855/jidc.829

[9] Hafner J , Tuma F, Hoilat GJ et al Intestinal perforation. In: StatPearls. Treasure Island, FL: StatPearls Publishing, 2021. https://www.ncbi.nlm.nih.gov/books/NBK538191/ (9 February 2021, date last accessed). 30855779

[10] Cappell MS , Batke M. Mechanical obstruction of the small bowel and colon. Med Clin North Am 2008;92:575–97, viii. 18387377 10.1016/j.mcna.2008.01.003

[11] Loftus EV Jr . Clinical epidemiology of inflammatory bowel disease: incidence, prevalence, and environmental influences. Gastroenterology 2004;126:1504–17. 15168363 10.1053/j.gastro.2004.01.063

[12] Mahajan G , Kotru M, Sharma R et al Usefulness of histopathological examination in nontraumatic perforation of small intestine. J Gastrointest Surg 2011;15:1837–41. 21822757 10.1007/s11605-011-1646-z

[13] Welch JP , Donaldson GA. Perforative carcinoma of colon and rectum. Ann Surg 1974;180:734–40. 4423043 10.1097/00000658-197411000-00005 PMC1343685

[14] Zielinski MD , Merchea A, Heller SF et al Emergency management of perforated colon cancers: how aggressive should we be? J Gastrointest Surg 2011;15:2232–8. 21913040 10.1007/s11605-011-1674-8

[15] Chen TM , Huang YT, Wang GC. Outcome of colon cancer initially presenting as colon perforation and obstruction. World J Surg Onc 2017;15:164. 10.1186/s12957-017-1228-y PMC5574146 28841901

[16] Shin R , Lee SM, Sohn B et al Predictors of morbidity and mortality after surgery for intestinal perforation. Ann Coloproctol 2016;32:221–7. 28119865 10.3393/ac.2016.32.6.221 PMC5256250

[17] Tan KK , Bang SL, Sim R. Surgery for small bowel perforation in an Asian population: predictors of morbidity and mortality. J Gastrointest Surg 2010;14:493–9. 19997984 10.1007/s11605-009-1097-y

[18] Akinwale MO , Sanusi AA, Adebayo OK. Typhoid perforation: post-operative intensive care unit care and outcome. Afr J Paediatr Surg 2016;13:175–80. 28051046 10.4103/0189-6725.194664 PMC5154222

[19] Power GS , Harrison DA. Why try to predict ICU outcomes? Curr Opin Crit Care 2014;20:544–9. 25159474 10.1097/MCC.0000000000000136

[20] Dindo D , Demartines N, Clavien PA. Classification of surgical complications: a new proposal with evaluation in a cohort of 6336 patients and results of a survey. Ann Surg 2004;240:205–13. 15273542 10.1097/01.sla.0000133083.54934.ae PMC1360123

[21] Katayama H , Kurokawa Y, Nakamura K et al Extended Clavien-Dindo classification of surgical complications: Japan Clinical Oncology Group postoperative complications criteria. Surg Today 2016;46:668–85. 26289837 10.1007/s00595-015-1236-x PMC4848327

[22] Zhang S , Zheng C, Chen Y et al Clinicopathologic features, surgical treatments, and outcomes of small bowel tumors: a retrospective study in China. Int J Surg 2017;43:145–54. 28583893 10.1016/j.ijsu.2017.05.076

[23] Catena F , Ansaloni L, Gazzotti F et al Small bowel tumours in emergency surgery: specificity of clinical presentation. ANZ J Surg 2005;75:997–9. 16336396 10.1111/j.1445-2197.2005.03590.x

[24] Vaidya R , Habermann TM, Donohue JH et al Bowel perforation in intestinal lymphoma: incidence and clinical features. Ann Oncol 2013;24:2439–43. 23704194 10.1093/annonc/mdt188 PMC3755328

[25] Wada M , Onda M, Tokunaga A et al Spontaneous gastrointestinal perforation in patients with lymphoma receiving chemotherapy and steroids: report of three cases. Nihon Ika Daigaku Zasshi 1999;66:37–40. 10097589 10.1272/jnms.66.37

[26] Contini S. Typhoid intestinal perforation in developing countries: still unavoidable deaths? World J Gastroenterol 2017;23:1925–31. 28373758 10.3748/wjg.v23.i11.1925 PMC5360633

[27] Qi W-X , Shen Z, Tang L-N et al Bevacizumab increases the risk of gastrointestinal perforation in cancer patients: a meta-analysis with a focus on different subgroups. Eur J Clin Pharmacol 2014;70:893–906. 24858820 10.1007/s00228-014-1687-9

[28] Roodhart JM , Langenberg MH, Witteveen E et al The molecular basis of class side effects due to treatment with inhibitors of the VEGF/VEGFR pathway. Curr Clin Pharmacol 2008;3:132–43. 18690886 10.2174/157488408784293705

[29] Suzuki N , Tajiri K, Futsukaichi Y et al Perforation of the small intestine after introduction of lenvatinib in a patient with advanced hepatocellular carcinoma. Case Rep Gastroenterol 2020;14:63–9. 32110202 10.1159/000505774 PMC7036537

[30] Sharma R , Ranjan V, Jain S et al A prospective study evaluating utility of Mannheim peritonitis index in predicting prognosis of perforation peritonitis. J Nat Sc Biol Med 2015;6:49–52. 26604619 10.4103/0976-9668.166076 PMC4630763

[31] Singer M , Deutschman CS, Seymour CW et al The third international consensus definitions for sepsis and septic shock (Sepsis-3). JAMA 2016;315:801–10. 26903338 10.1001/jama.2016.0287 PMC4968574

[32] Churpek MM , Zadravecz FJ, Winslow C et al Incidence and prognostic value of the systemic inflammatory response syndrome and organ dysfunctions in ward patients. Am J Respir Crit Care Med 2015;192:958–64. 26158402 10.1164/rccm.201502-0275OC PMC4642209

[33] Seymour CW , Liu VX, Iwashyna TJ et al Assessment of clinical criteria for sepsis: for the third international consensus definitions for sepsis and septic shock (Sepsis-3). JAMA 2016;315:762–74. 26903335 10.1001/jama.2016.0288 PMC5433435

[34] Horiuchi A , Watanabe Y, Doi T et al Evaluation of prognostic factors and scoring system in colonic perforation. World J Gastroenterol 2007;13:3228–31. 17589902 10.3748/wjg.v13.i23.3228 PMC4436609

[35] Shimazaki J , Motohashi G, Nishida K et al Postoperative arterial blood lactate level as a mortality marker in patients with colorectal perforation. Int J Colorectal Dis 2014;29:51–5. 23846515 10.1007/s00384-013-1738-1 PMC3898377

[36] Jain S , Sinha S, Sharma SK et al Procalcitonin as a prognostic marker for sepsis: a prospective observational study. BMC Res Notes 2014;7:458. 25034373 10.1186/1756-0500-7-458 PMC4105100

[37] Jekarl DW , Lee S, Kim M et al Procalcitonin as a prognostic marker for sepsis based on SEPSIS-3. J Clin Lab Anal 2019;33:e22996. 31420921 10.1002/jcla.22996 PMC6868407

[38] Schuetz P , Birkhahn R, Sherwin R et al Serial procalcitonin predicts mortality in severe sepsis patients: results from the multicenter procalcitonin MOnitoring SEpsis (MOSES) Study. Crit Care Med 2017;45:781–9. 28257335 10.1097/CCM.0000000000002321 PMC5389588

[39] Maseda E , Suarez-DE-LA-Rica A, Anillo V et al Procalcitonin-guided therapy may reduce length of antibiotic treatment in intensive care unit patients with secondary peritonitis: a multicenter retrospective study. J Crit Care 2015;30:537–42. 25600574 10.1016/j.jcrc.2014.12.014

[40] Bloos F , Trips E, Nierhaus A et al; for SepNet Critical Care Trials Group. Effect of sodium selenite administration and procalcitonin-guided therapy on mortality in patients with severe sepsis or septic shock: a randomized clinical trial. JAMA Intern Med 2016;176:1266–76. 27428731 10.1001/jamainternmed.2016.2514

[41] Jekarl DW , Kim KS, Lee S et al Cytokine and molecular networks in sepsis cases: a network biology approach. Eur Cytokine Netw 2018;29:103–11. 30547887 10.1684/ecn.2018.0414

