# Supplementary material for: Predictors of mortality in patients with acute small-bowel perforation transferred to ICU after emergency surgery: a single-centre retrospective cohort study
Source: Gastroenterol Rep (Oxf). 2021 Dec 28;10:goab054. doi: 10.1093/gastro/goab054 (PMC8972993; doi:10.1093/gastro/goab054)
Supplement: goab054_Supplementary_Data [file goab054_supplementary_data.zip › Supplementary.docx]

**Supplementary material**

**Supplementary Table 1.** Histopathologic distribution of 24 cases of lymphoma responsible for small bowel perforation

| **Histopathologic type** | **No. of patients (%)** |
| --- | --- |
| B-cell type | 10 (41.7) |
| Follicular, grade 1 | 1 (4.17) |
| Diffuse large B-cell | 9 (37.5) |
| T-cell type | 14 (58.3) |
| MEITL | 11 (45.8) |
| EATL | 1 (4.17) |
| NK/T-cell | 1 (4.17) |
| Systemic EBV^+^ T-cell lymphoma | 1 (4.17) |

EATL, Enteropathy-type T-cell lymphoma; EBV, Epstein-Barr virus; MEITL, Monomorphic epitheliotropic intestinal T-cell lymphoma; NK, Natural killer.
