# Supplementary figures and images for: Predictors of mortality in patients with acute small-bowel perforation transferred to ICU after emergency surgery: a single-centre retrospective cohort study
Source: Gastroenterol Rep (Oxf). 2021 Dec 28;10:goab054. doi: 10.1093/gastro/goab054 (PMC8972993; doi:10.1093/gastro/goab054)

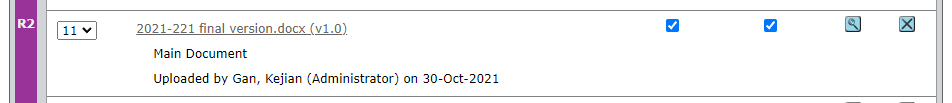

Supplement: goab054_Supplementary_Data [file goab054_supplementary_data.zip › 1.png]
